# Diethyl [(5-chloro-2-hydroxy­anilino)(4-chloro­phen­yl)meth­yl]phospho­nate

**DOI:** 10.1107/S1600536809037039

**Published:** 2009-09-19

**Authors:** M. Krishnaiah, V. H. H. Surendra Babu, G. Syam Prasad, C. Suresh Reddy, Vedavati G. Puranik

**Affiliations:** a Department of Physics, S.V. University, Tirupati 517 502, India; b Department of Chemistry, S.V. University, Tirupati 517 502, India; c Centre of Material Characterization, National Chemical Laboratory, Pune 411 008, India

## Abstract

In the title compound, C_17_H_20_Cl_2_NO_4_P, the P atom is bonded in a distorted tetra­hedral environment. The dihedral angle between the two benzene rings is 80.5 (1)°. In the crystal structure, inter­molecular O—H⋯O and N—H⋯O hydrogen bonds link pairs of mol­ecules into centrosymmetric dimers. These dimers, are in turn, linked by weak inter­molecular C—H⋯O hydrogen bonds into one-dimensional chains along [010]. Additional stabilization is provided by very weak C—H⋯Cl inter­actions.

## Related literature

For applications of α-amino­phospho­nates, see: Allen *et al.* (1989 ▶); Baylis *et al.* (1984 ▶); Fields (1999 ▶); Hirschmann *et al.* (1994 ▶); Kafarski & Lejczak (1991 ▶); Miliszkiewicz *et al.* (1992 ▶). For the anti­bacterial activity of the title compound, see: Syam Prasad *et al.* (2007 ▶). For related structures, see: Boehlow *et al.* (1997 ▶); Yang *et al.* (2005 ▶); Sawka-Dobrowolska & Kowalik (1985 ▶); Sawka-Dobrowolska & Rułko (1987 ▶); Sanders *et al.* (1996 ▶); Ezra & Collin (1973 ▶). For P—C bond lengths in related structures, see: Rużić-Toroš *et al.* (1978 ▶).
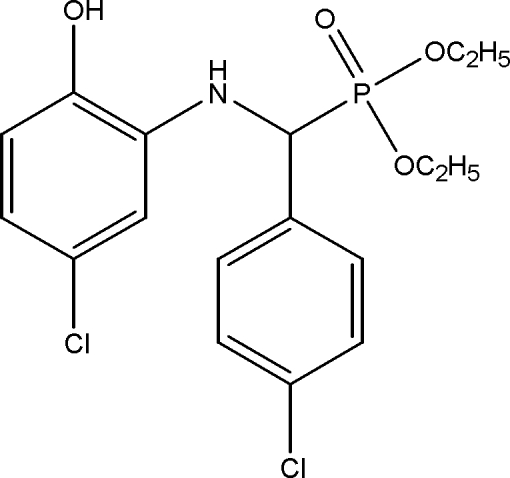


## Experimental

### 

#### Crystal data

C_17_H_20_Cl_2_NO_4_P
*M* *_r_* = 404.21
Triclinic, 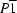

*a* = 7.790 (3) Å
*b* = 9.297 (4) Å
*c* = 14.372 (6) Å
α = 82.817 (6)°
β = 80.842 (6)°
γ = 70.323 (6)°
*V* = 964.7 (7) Å^3^
*Z* = 2
Mo *K*α radiation
μ = 0.44 mm^−1^
*T* = 294 K
0.25 × 0.25 × 0.13 mm

#### Data collection

Siemens SMART CCD area-detector diffractometer
Absorption correction: multi-scan (*SADABS*; Sheldrick, 1996 ▶) *T*_min_ = 0.896, *T*_max_ = 0.944
10997 measured reflections
4863 independent reflections
3635 reflections with *I* > 2σ(*I*)
*R*_int_ = 0.017

#### Refinement

*R*[*F* ^2^ > 2σ(*F* ^2^)] = 0.053
*wR*(*F* ^2^) = 0.159
*S* = 1.05
4863 reflections
226 parameters
H-atom parameters constrained
Δρ_max_ = 0.56 e Å^−3^
Δρ_min_ = −0.34 e Å^−3^

### 

Data collection: *SMART* (Bruker 2001 ▶); cell refinement: *SAINT* (Bruker 2002 ▶); data reduction: *SAINT*; program(s) used to solve structure: *SHELXS97* (Sheldrick, 2008 ▶); program(s) used to refine structure: *SHELXL97* (Sheldrick, 2008 ▶); molecular graphics: *ZORTEPII* (Zsolnai, 1997 ▶) and *PLATON* (Spek, 2009 ▶); software used to prepare material for publication: *enCIFer* (Allen *et al.*, 2004 ▶) and *PARST* (Nardelli, 1995 ▶).

## Supplementary Material

Crystal structure: contains datablocks global, I. DOI: 10.1107/S1600536809037039/lh2891sup1.cif

Structure factors: contains datablocks I. DOI: 10.1107/S1600536809037039/lh2891Isup2.hkl

Additional supplementary materials:  crystallographic information; 3D view; checkCIF report

## Figures and Tables

**Table 1:** Hydrogen-bond geometry (Å, °)

*D*—H⋯*A*	*D*—H	H⋯*A*	*D*⋯*A*	*D*—H⋯*A*
N4—H4⋯O8^i^	0.86	2.48	3.286 (3)	157
C24—H24*B*⋯O5^ii^	0.97	2.57	3.504 (4)	162
O8—H8⋯O5^i^	0.82	1.92	2.636 (2)	145
C15—H15⋯Cl2^iii^	0.98	2.91	3.872 (3)	165
N4—H4⋯O8	0.86	2.28	2.634 (3)	105

Symmetry codes: (i) 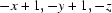; (ii) 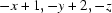; (iii) 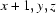.

**Fig. 1.**
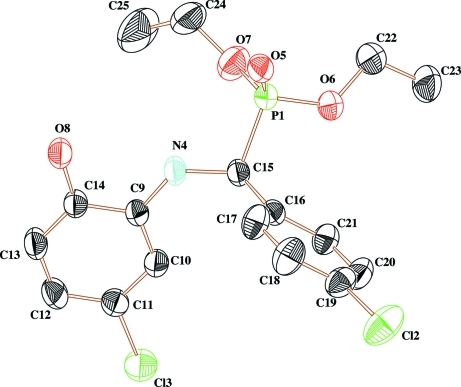
The molecular structure of the itle compound with displacement ellipsoids drawn at the 40% probability level. H atoms have been omitted.

**Fig. 2.**
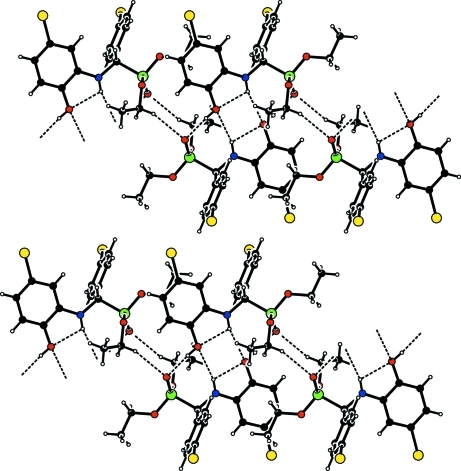
Part of the crystal structure showing one-dimensional chains along [010]. Hydrogen bonds are shown as dashed lines.

## supplementary crystallographic information

### Comment

The synthesis of α-aminophosphonates has attracted much interest because of their biological activity and structural analogy to α-amino acids (Fields, 1999). They also act as peptide mimics (Kafarski *et al.*, 1991), enzyme inhibitors (Allen *et al.*, 1989), haptens of catalytic antibodies (Hirschmann *et al.*, 1994), antibiotics and pharmacological agents (Baylis *et al.*, 1984) and the analogues of amino acids, aminophosphonic and aminophophinic acids as plant growth regulators and herbicides (Miliszkiewicz *et al.*, 1992). As a result, a variety of synthetic approaches have been developed for the synthesis of α-aminophosphonates. The title compound (I) exhibits antibacterial activity against Gram positive as well as Gram negative bacteria (Syam *et al.*, 2007)

The molecular structure of the title compound is shown in Fig. 1. (I). The P—O bond distances are in good agreement with those in related structures (Boehlow *et al.*, 1997; Yang *et al.*, 2005). The P1—C15 bond length is in good agreement with the reported values of 1.821 (6) Å, 1.808 (9) Å, 1.813 (6)Å in related structures (Rużić-Toroš *et al.*, 1978; Sawka-Dobrowolska & Rułko, 1987; Sanders *et al.*, 1996). The C9–N4 bond length (1.384 (2) Å) is intermediate between C—N and C=N double bond distances, which shows influence of the delocalization of electrons from the benzene ring. The P atom adopts a distorted tetrahedral configuration, with the angles in the range of 101.1 (1)° - 115.8 (1)°. The deformation of the PO3C tetrahedra may be explained in terms of steric effects from the adjacent bulky ethoxy groups. There are some differences between similar bonds in the P-O-C-C groups and this may be attributed to the larger than normal anisotropic displacement parameters of the atoms in the groups. This was not considered severe enough to model as disorder (Ezra & Collin, 1973; Sawka-Dobrowolska & Kowalik, 1985; Sanders *et al.*, 1996). In the crystal structure, intermolecular O-H···O and N-H···O hydrogen bonds link pairs of molecules into centrosymmetric dimers. These dimers, are in turn, linked by weak intermolecular C-H···O hydrogen bonds into one-dimensional chains along [010](see Fig. 2). Additional stabilization is provided by very weak C—H···Cl interactions. .

### Experimental

To a stirred solution of 2-amino-4-chlorophenol (0.72 g, 0.005 mol), 4-chloro benzaldehyde (0.005 mol) in anhydrous toluene (15 ml) was added dropwise. Stirring was continued at room temperature for lh. Then diethylphosphite (0.7 g, 0.005 mol) in anhydrous toulene (15 ml) was added dropwise. Stirring was continued at room temperature for another 0.5 h, later the mixture was heated under reflux for 4–6 h. After completion of the reaction (monitored by TLC) the solvent was removed under reduced pressure. The resulting residue was purified by column chromatography on silica gel using petroleum ether-ethyl acetate (8:2) as eluent. Colorless, prism shaped single crystals suitable for diffraction studies were grown by slow evaporation of a methanol solution of the title compound.

### Refinement

All hydrogen atoms were placed in calculated positions with C-H = 0.93-0.97Å; N-H = 0.86Å and O-H = 0.82Å. They were included in the refinement in a riding-model approximation with U_iso_(H) = 1.2U_eq_(C,N) or 1.5U_eq_(C_methyl_,O)

### Crystal data

**Table:**

C_17_H_20_Cl_2_NO_4_P	*Z* = 2
*M**_r_* = 404.21	*F*(000) = 420
Triclinic, *P*1	*D*_x_ = 1.391 Mg m^−^^3^*D*_m_ = 1.390 Mg m^−^^3^*D*_m_ measured by not measured
Hall symbol: -P 1	Mo *K*α radiation, λ = 0.71073 Å
*a* = 7.790 (3) Å	Cell parameters from 4863 reflections
*b* = 9.297 (4) Å	θ = 2.3–28.4°
*c* = 14.372 (6) Å	µ = 0.44 mm^−^^1^
α = 82.817 (6)°	*T* = 294 K
β = 80.842 (6)°	Prism, colorless
γ = 70.323 (6)°	0.25 × 0.25 × 0.13 mm
*V* = 964.7 (7) Å^3^	

### Data collection

**Table:**

Siemens SMART CCD area-detector diffractometer	4863 independent reflections
Radiation source: fine-focus sealed tube	3635 reflections with *I* > 2σ(*I*)
graphite	*R*_int_ = 0.017
ω scans	θ_max_ = 28.4°, θ_min_ = 2.3°
Absorption correction: multi-scan (*SADABS*; Sheldrick, 1996)	*h* = −10→10
*T*_min_ = 0.896, *T*_max_ = 0.944	*k* = −12→12
10997 measured reflections	*l* = −19→19

### Refinement

**Table:**

Refinement on *F*^2^	Primary atom site location: structure-invariant direct methods
Least-squares matrix: full	Secondary atom site location: difference Fourier map
*R*[*F*^2^ > 2σ(*F*^2^)] = 0.053	Hydrogen site location: inferred from neighbouring sites
*wR*(*F*^2^) = 0.159	H-atom parameters constrained
*S* = 1.05	*w* = 1/[σ^2^(*F*_o_^2^) + (0.0937*P*)^2^ + 0.273*P*] where *P* = (*F*_o_^2^ + 2*F*_c_^2^)/3
4863 reflections	(Δ/σ)_max_ < 0.001
226 parameters	Δρ_max_ = 0.56 e Å^−^^3^
0 restraints	Δρ_min_ = −0.34 e Å^−^^3^

### Special details

**Table:**

Geometry. All e.s.d.'s (except the e.s.d. in the dihedral angle between two l.s. planes) are estimated using the full covariance matrix. The cell e.s.d.'s are taken into account individually in the estimation of e.s.d.'s in distances, angles and torsion angles; correlations between e.s.d.'s in cell parameters are only used when they are defined by crystal symmetry. An approximate (isotropic) treatment of cell e.s.d.'s is used for estimating e.s.d.'s involving l.s. planes.
Refinement. Refinement of *F*^2^ against ALL reflections. The weighted *R*-factor *wR* and goodness of fit *S* are based on *F*^2^, conventional *R*-factors *R* are based on *F*, with *F* set to zero for negative *F*^2^. The threshold expression of *F*^2^ > σ(*F*^2^) is used only for calculating *R*-factors(gt) *etc*. and is not relevant to the choice of reflections for refinement. *R*-factors based on *F*^2^ are statistically about twice as large as those based on *F*, and *R*- factors based on ALL data will be even larger.

### Fractional atomic coordinates and isotropic or equivalent isotropic displacement parameters (Å^2^)

**Table:**

	*x*	*y*	*z*	*U*_iso_*/*U*_eq_	
P1	0.39158 (8)	0.81933 (6)	0.17033 (4)	0.05033 (18)	
Cl3	0.76222 (12)	0.10544 (8)	0.42621 (5)	0.0856 (3)	
Cl2	−0.32728 (10)	0.63628 (13)	0.43963 (6)	0.0973 (3)	
O8	0.6784 (2)	0.33466 (17)	0.03123 (10)	0.0610 (4)	
H8	0.7402	0.2713	−0.0061	0.092*	
C15	0.4115 (3)	0.6327 (2)	0.23264 (13)	0.0462 (4)	
H15	0.4946	0.6167	0.2803	0.055*	
O5	0.2676 (2)	0.86066 (17)	0.09700 (11)	0.0619 (4)	
C16	0.2269 (3)	0.6284 (2)	0.28494 (13)	0.0449 (4)	
N4	0.4997 (3)	0.51803 (19)	0.16550 (12)	0.0555 (5)	
H4	0.4846	0.5413	0.1069	0.067*	
C10	0.6279 (3)	0.3174 (2)	0.28709 (14)	0.0506 (5)	
H10	0.5652	0.3800	0.3356	0.061*	
C14	0.7033 (3)	0.2733 (2)	0.12175 (14)	0.0474 (4)	
O7	0.5926 (3)	0.8172 (2)	0.13357 (14)	0.0790 (5)	
C9	0.6079 (2)	0.3719 (2)	0.19319 (14)	0.0435 (4)	
O6	0.3313 (3)	0.92357 (18)	0.25403 (11)	0.0662 (5)	
C11	0.7415 (3)	0.1695 (2)	0.30794 (15)	0.0550 (5)	
C13	0.8153 (3)	0.1274 (3)	0.14481 (17)	0.0600 (6)	
H13	0.8783	0.0637	0.0969	0.072*	
C12	0.8360 (3)	0.0736 (3)	0.23795 (18)	0.0634 (6)	
H12	0.9121	−0.0251	0.2529	0.076*	
C21	0.1714 (3)	0.6774 (3)	0.37545 (15)	0.0567 (5)	
H21	0.2495	0.7088	0.4048	0.068*	
C19	−0.1119 (3)	0.6310 (3)	0.38023 (17)	0.0622 (6)	
C20	0.0014 (3)	0.6800 (3)	0.42263 (16)	0.0646 (6)	
H20	−0.0357	0.7150	0.4828	0.078*	
C17	0.1099 (3)	0.5794 (3)	0.24381 (17)	0.0646 (6)	
H17	0.1460	0.5448	0.1835	0.078*	
C18	−0.0595 (4)	0.5810 (4)	0.2904 (2)	0.0754 (7)	
H18	−0.1376	0.5487	0.2616	0.091*	
C22	0.3166 (5)	1.0826 (3)	0.2469 (2)	0.0840 (9)	
H22A	0.2271	1.1403	0.2048	0.101*	
H22B	0.4343	1.0945	0.2209	0.101*	
C24	0.6719 (5)	0.8128 (4)	0.0351 (2)	0.0952 (10)	
H24A	0.5890	0.7947	−0.0023	0.114*	
H24B	0.6872	0.9110	0.0124	0.114*	
C23	0.2602 (5)	1.1411 (4)	0.3403 (2)	0.0903 (10)	
H23A	0.2494	1.2477	0.3353	0.135*	
H23B	0.3504	1.0850	0.3813	0.135*	
H23C	0.1437	1.1292	0.3657	0.135*	
C25	0.8425 (6)	0.6970 (6)	0.0239 (3)	0.148 (2)	
H25A	0.8922	0.6963	−0.0417	0.222*	
H25B	0.8271	0.5996	0.0455	0.222*	
H25C	0.9252	0.7158	0.0601	0.222*	

### Atomic displacement parameters (Å^2^)

**Table:**

	*U*^11^	*U*^22^	*U*^33^	*U*^12^	*U*^13^	*U*^23^
P1	0.0602 (3)	0.0418 (3)	0.0429 (3)	−0.0107 (2)	0.0019 (2)	−0.0079 (2)
Cl3	0.1068 (6)	0.0725 (4)	0.0536 (4)	0.0006 (4)	−0.0165 (3)	0.0075 (3)
Cl2	0.0603 (4)	0.1562 (8)	0.0703 (5)	−0.0335 (4)	0.0017 (3)	−0.0068 (5)
O8	0.0729 (10)	0.0504 (8)	0.0449 (8)	0.0014 (7)	−0.0067 (7)	−0.0104 (6)
C15	0.0516 (10)	0.0384 (9)	0.0407 (9)	−0.0028 (8)	−0.0057 (8)	−0.0070 (7)
O5	0.0798 (11)	0.0474 (8)	0.0463 (8)	−0.0043 (7)	−0.0086 (7)	−0.0038 (6)
C16	0.0524 (10)	0.0355 (8)	0.0401 (9)	−0.0045 (7)	−0.0078 (8)	−0.0038 (7)
N4	0.0666 (11)	0.0432 (9)	0.0396 (8)	0.0062 (8)	−0.0066 (8)	−0.0081 (7)
C10	0.0520 (11)	0.0473 (10)	0.0449 (10)	−0.0069 (8)	−0.0017 (8)	−0.0071 (8)
C14	0.0460 (10)	0.0457 (10)	0.0455 (10)	−0.0072 (8)	−0.0037 (8)	−0.0088 (8)
O7	0.0733 (11)	0.0911 (13)	0.0722 (12)	−0.0324 (10)	0.0114 (9)	−0.0145 (10)
C9	0.0404 (9)	0.0386 (9)	0.0463 (10)	−0.0062 (7)	−0.0030 (7)	−0.0062 (7)
O6	0.0935 (12)	0.0494 (8)	0.0552 (9)	−0.0257 (8)	0.0070 (8)	−0.0162 (7)
C11	0.0576 (12)	0.0496 (11)	0.0498 (11)	−0.0071 (9)	−0.0092 (9)	−0.0002 (9)
C13	0.0604 (12)	0.0482 (11)	0.0565 (12)	0.0038 (9)	−0.0036 (10)	−0.0140 (9)
C12	0.0623 (13)	0.0452 (11)	0.0655 (14)	0.0060 (9)	−0.0101 (11)	−0.0042 (10)
C21	0.0623 (13)	0.0642 (13)	0.0442 (11)	−0.0187 (10)	−0.0053 (9)	−0.0129 (9)
C19	0.0513 (11)	0.0742 (15)	0.0531 (12)	−0.0124 (10)	−0.0051 (9)	0.0004 (11)
C20	0.0677 (14)	0.0764 (15)	0.0436 (11)	−0.0162 (12)	0.0013 (10)	−0.0124 (10)
C17	0.0645 (13)	0.0807 (16)	0.0500 (12)	−0.0205 (12)	−0.0052 (10)	−0.0212 (11)
C18	0.0658 (15)	0.103 (2)	0.0634 (15)	−0.0306 (14)	−0.0073 (12)	−0.0221 (14)
C22	0.120 (2)	0.0619 (15)	0.0744 (17)	−0.0419 (16)	0.0128 (16)	−0.0183 (13)
C24	0.084 (2)	0.097 (2)	0.079 (2)	−0.0163 (17)	0.0210 (16)	0.0078 (17)
C23	0.119 (3)	0.0762 (18)	0.083 (2)	−0.0425 (18)	0.0148 (18)	−0.0354 (15)
C25	0.090 (3)	0.164 (4)	0.123 (4)	0.017 (3)	0.034 (2)	0.008 (3)

### Geometric parameters (Å, °)

**Table:**

P1—O5	1.4712 (17)	C13—C12	1.384 (3)
P1—O6	1.5531 (16)	C13—H13	0.9300
P1—O7	1.565 (2)	C12—H12	0.9300
P1—C15	1.819 (2)	C21—C20	1.382 (3)
Cl3—C11	1.743 (2)	C21—H21	0.9300
Cl2—C19	1.745 (3)	C19—C20	1.367 (4)
O8—C14	1.370 (2)	C19—C18	1.380 (4)
O8—H8	0.8200	C20—H20	0.9300
C15—N4	1.447 (2)	C17—C18	1.377 (4)
C15—C16	1.523 (3)	C17—H17	0.9300
C15—H15	0.9800	C18—H18	0.9300
C16—C17	1.381 (3)	C22—C23	1.457 (4)
C16—C21	1.387 (3)	C22—H22A	0.9700
N4—C9	1.384 (2)	C22—H22B	0.9700
N4—H4	0.8600	C24—C25	1.402 (5)
C10—C11	1.388 (3)	C24—H24A	0.9700
C10—C9	1.394 (3)	C24—H24B	0.9700
C10—H10	0.9300	C23—H23A	0.9600
C14—C13	1.376 (3)	C23—H23B	0.9600
C14—C9	1.407 (3)	C23—H23C	0.9600
O7—C24	1.450 (4)	C25—H25A	0.9600
O6—C22	1.436 (3)	C25—H25B	0.9600
C11—C12	1.377 (3)	C25—H25C	0.9600
			
O5—P1—O6	115.79 (10)	C20—C21—C16	120.8 (2)
O5—P1—O7	114.61 (11)	C20—C21—H21	119.6
O6—P1—O7	104.38 (10)	C16—C21—H21	119.6
O5—P1—C15	113.51 (10)	C20—C19—C18	120.8 (2)
O6—P1—C15	101.05 (9)	C20—C19—Cl2	119.40 (19)
O7—P1—C15	106.08 (11)	C18—C19—Cl2	119.8 (2)
C14—O8—H8	109.5	C19—C20—C21	119.6 (2)
N4—C15—C16	115.71 (17)	C19—C20—H20	120.2
N4—C15—P1	107.67 (14)	C21—C20—H20	120.2
C16—C15—P1	111.24 (12)	C18—C17—C16	121.3 (2)
N4—C15—H15	107.3	C18—C17—H17	119.4
C16—C15—H15	107.3	C16—C17—H17	119.4
P1—C15—H15	107.3	C17—C18—C19	119.2 (2)
C17—C16—C21	118.4 (2)	C17—C18—H18	120.4
C17—C16—C15	121.66 (18)	C19—C18—H18	120.4
C21—C16—C15	119.92 (18)	O6—C22—C23	109.6 (2)
C9—N4—C15	121.70 (16)	O6—C22—H22A	109.8
C9—N4—H4	119.1	C23—C22—H22A	109.8
C15—N4—H4	119.1	O6—C22—H22B	109.8
C11—C10—C9	119.82 (18)	C23—C22—H22B	109.8
C11—C10—H10	120.1	H22A—C22—H22B	108.2
C9—C10—H10	120.1	C25—C24—O7	111.0 (3)
O8—C14—C13	124.40 (18)	C25—C24—H24A	109.4
O8—C14—C9	115.33 (17)	O7—C24—H24A	109.4
C13—C14—C9	120.25 (19)	C25—C24—H24B	109.4
C24—O7—P1	124.7 (2)	O7—C24—H24B	109.4
N4—C9—C10	123.96 (17)	H24A—C24—H24B	108.0
N4—C9—C14	117.57 (17)	C22—C23—H23A	109.5
C10—C9—C14	118.47 (17)	C22—C23—H23B	109.5
C22—O6—P1	125.27 (17)	H23A—C23—H23B	109.5
C12—C11—C10	121.7 (2)	C22—C23—H23C	109.5
C12—C11—Cl3	119.93 (17)	H23A—C23—H23C	109.5
C10—C11—Cl3	118.42 (17)	H23B—C23—H23C	109.5
C14—C13—C12	121.27 (19)	C24—C25—H25A	109.5
C14—C13—H13	119.4	C24—C25—H25B	109.5
C12—C13—H13	119.4	H25A—C25—H25B	109.5
C11—C12—C13	118.53 (19)	C24—C25—H25C	109.5
C11—C12—H12	120.7	H25A—C25—H25C	109.5
C13—C12—H12	120.7	H25B—C25—H25C	109.5
			
O5—P1—C15—N4	65.76 (17)	O5—P1—O6—C22	−65.0 (3)
O6—P1—C15—N4	−169.61 (14)	O7—P1—O6—C22	62.0 (3)
O7—P1—C15—N4	−60.97 (16)	C15—P1—O6—C22	172.0 (2)
O5—P1—C15—C16	−62.00 (16)	C9—C10—C11—C12	−0.2 (4)
O6—P1—C15—C16	62.62 (15)	C9—C10—C11—Cl3	−179.79 (16)
O7—P1—C15—C16	171.27 (13)	O8—C14—C13—C12	179.0 (2)
N4—C15—C16—C17	−30.9 (3)	C9—C14—C13—C12	0.5 (4)
P1—C15—C16—C17	92.4 (2)	C10—C11—C12—C13	−0.2 (4)
N4—C15—C16—C21	150.44 (18)	Cl3—C11—C12—C13	179.4 (2)
P1—C15—C16—C21	−86.3 (2)	C14—C13—C12—C11	0.1 (4)
C16—C15—N4—C9	−84.7 (2)	C17—C16—C21—C20	−1.3 (3)
P1—C15—N4—C9	150.18 (17)	C15—C16—C21—C20	177.43 (19)
O5—P1—O7—C24	−16.7 (3)	C18—C19—C20—C21	−1.0 (4)
O6—P1—O7—C24	−144.4 (2)	Cl2—C19—C20—C21	−179.39 (19)
C15—P1—O7—C24	109.4 (2)	C16—C21—C20—C19	1.3 (4)
C15—N4—C9—C10	6.3 (3)	C21—C16—C17—C18	1.0 (4)
C15—N4—C9—C14	−173.55 (18)	C15—C16—C17—C18	−177.7 (2)
C11—C10—C9—N4	−179.1 (2)	C16—C17—C18—C19	−0.7 (4)
C11—C10—C9—C14	0.7 (3)	C20—C19—C18—C17	0.7 (4)
O8—C14—C9—N4	0.3 (3)	Cl2—C19—C18—C17	179.1 (2)
C13—C14—C9—N4	179.0 (2)	P1—O6—C22—C23	−178.8 (2)
O8—C14—C9—C10	−179.55 (19)	P1—O7—C24—C25	−130.1 (4)
C13—C14—C9—C10	−0.9 (3)		

### Hydrogen-bond geometry (Å, °)

**Table:**

*D*—H···*A*	*D*—H	H···*A*	*D*···*A*	*D*—H···*A*
N4—H4···O8^i^	0.86	2.48	3.286 (3)	157
C24—H24B···O5^ii^	0.97	2.57	3.504 (4)	162
O8—H8···O5^i^	0.82	1.92	2.636 (2)	145
C15—H15···Cl2^iii^	0.98	2.91	3.872 (3)	165
N4—H4···O8	0.86	2.28	2.634 (3)	105
C24—H24A···O5	0.97	2.59	3.029 (5)	107

Symmetry codes: (i) −*x*+1, −*y*+1, −*z*; (ii) −*x*+1, −*y*+2, −*z*; (iii) *x*+1, *y*, *z*.
